# Supplementary material for: Cowpox Viruses: A Zoo Full of Viral Diversity and Lurking Threats
Source: Biomolecules. 2023 Feb 8;13(2):325. doi: 10.3390/biom13020325 (PMC9953750; doi:10.3390/biom13020325)
Supplement: Supplementary file 1 [file biomolecules-13-00325-s001.zip › Supplementary file S3 image rights.docx]

**Cowpox Viruses: A Zoo Full of Viral Diversity and Lurking Threats**

**Image References, Rights, and Licenses**

**Figure 1 Timeline of major events in cowpox virus history**

**165:**

*The angel of death striking a door during the plague of Rome*. Engraving by Levasseur after J. Delaunay.Image sourced from [Wellcome Images](http://wellcomeimages.org/), This file is licensed under the [Creative Commons](https://en.wikipedia.org/wiki/en:Creative_Commons) [Attribution 4.0 International](https://creativecommons.org/licenses/by/4.0/deed.en) license. https://commons.wikimedia.org/wiki/File:The_angel_of_death_striking_a_door_during_the_plague_of_Rome_Wellcome_V0010664.jpg

**1000s:**

*Relief in front of the Asakura Ishikai-Hospital commemorating the introduction of the smallpox variolation by the Japanese physician Ogata Shunsaku (1748-1810)*. [Creative Commons](https://en.wikipedia.org/wiki/en:Creative_Commons) [CC0 1.0 Universal Public Domain Dedication](https://creativecommons.org/publicdomain/zero/1.0/deed.en) from user [WolfgangMichel](https://commons.wikimedia.org/wiki/User:WolfgangMichel) <https://commons.wikimedia.org/wiki/File:Ogata-Shunsaku-Relief-Amagi-Hospital.jpg>

**1500s:**

*Florentine Codex smallpox.* Date 23 January 2009 from Author [Jaontiveros](https://commons.wikimedia.org/wiki/User:Jaontiveros) This file is **licensed** under the [Creative Commons](https://en.wikipedia.org/wiki/en:Creative_Commons) Attribution-Share Alike [4.0 International](https://creativecommons.org/licenses/by-sa/4.0/), [3.0 Unported](https://creativecommons.org/licenses/by-sa/3.0/deed.en), [2.5 Generic](https://creativecommons.org/licenses/by-sa/2.5/deed.en), [2.0 Generic](https://creativecommons.org/licenses/by-sa/2.0/deed.en) and [1.0 Generic](https://creativecommons.org/licenses/by-sa/1.0/deed.en) license. https://commons.wikimedia.org/wiki/File:Florentinoviruela.JPG

**1720s:**

*A Woman Called Lady Mary Wortley Montagu*. Sir Godfrey Kneller - The author died in 1723, so this work is in the [**public domain**](https://en.wikipedia.org/wiki/public_domain) in its country of origin and other countries and areas where the [copyright term](https://en.wikipedia.org/wiki/List_of_countries%27_copyright_lengths) is the author's life plus 100 years or fewer. This work is in the [**public domain**](https://en.wikipedia.org/wiki/public_domain) in the [United States](https://en.wikipedia.org/wiki/United_States) because it was [published](https://commons.wikimedia.org/wiki/Commons:Publication) (or registered with the [U.S. Copyright Office](https://en.wikipedia.org/wiki/United_States_Copyright_Office)) before January 1, 1927. https://commons.wikimedia.org/wiki/File:Sir_Godfrey_Kneller_-_A_Woman_Called_Lady_Mary_Wortley_Montagu_-_Google_Art_Project.jpg Source: [PQFHiN79AfYWPg at Google Cultural Institute](https://www.google.com/culturalinstitute/asset-viewer/PQFHiN79AfYWPg)

**1768:**

A cow's udder with vaccinia pustules and human arms exhibiting both smallpox and cowpox pustules. Coloured engraving by J. Pass, 1811. <https://wellcomecollection.org/works/pfxyyrf6>

**License:** Public Domain

**1796:**

*Edward Jenner. Photograph of a sculpture by Giulio Monteverde*. This file comes from [Wellcome Images](http://wellcomeimages.org/), This file is licensed under the [Creative Commons](https://en.wikipedia.org/wiki/en:Creative_Commons) [Attribution 4.0 International](https://creativecommons.org/licenses/by/4.0/deed.en) license. https://commons.wikimedia.org/wiki/File:Edward_Jenner._Photograph_of_a_sculpture_by_Giulio_Monteverd_Wellcome_V0028722.jpg

**1800s:**

**Credit:** *Vaccine made from calf lymph*, London, England, 1956.

[Science Museum, London](https://wellcomecollection.org/works/hkkcm6nd). **Licence:** [Attribution 4.0 International (CC BY 4.0)](http://creativecommons.org/licenses/by/4.0/) <https://wellcomecollection.org/works/hkkcm6nd/images?id=jerjjnmk>

**1890s:**

Left (*Dmitry Iosifovich Ivanovsky*.jpg ) This work is in the [**public domain**](https://en.wikipedia.org/wiki/public_domain) in its country of origin and other countries and areas where the [copyright term](https://en.wikipedia.org/wiki/List_of_countries%27_copyright_lengths) is the author's life plus 70 years or fewer. https://commons.wikimedia.org/wiki/File:Dmitry_Iosifovich_Ivanovsky.jpg

Right (*Martinus Willem Beijerinck (1851-1931), Dutch microbiologist*.) Published in Proceeding of the Royal Society of London, 1931-1932

<https://wellcomeimages.org/indexplus/image/M0002348.html> Unknown author, This file is licensed under the [Creative Commons](https://en.wikipedia.org/wiki/en:Creative_Commons) [Attribution 4.0 International](https://creativecommons.org/licenses/by/4.0/deed.en) license.

**1938:**

*Allan Watt Downie* by Elliott & Fry bromide print, May 1955 NPG x89070

https://www.npg.org.uk/collections/search/use-this-image/?mkey=mw100640

**License terms**

Reproduction inside a journal or magazine, world, multiple languages provided that the publication is non-commercial in purpose, is not being sold to generate profit, and has a combined print-run/electronic total of not more than 4,000 copies. In addition to the print run/electronic total, permission includes posting the publication on an open access website, provided that access to the website and the publication is at no cost to the end user, for the life of the website.

Name: Non-commercial / Student thesis or dissertation terms. For reproduction within a thesis document submitted by a student at an educational establishment (an electronic version of the research may be stored online as long as it is made available at no cost to the end user) Project title: Chapter 1 Cowpox Viruses: A Zoo Full of Viral Diversity Dissertation for Ph.D. Immunology Ryan Bruneau

**Biomolecules license**

**
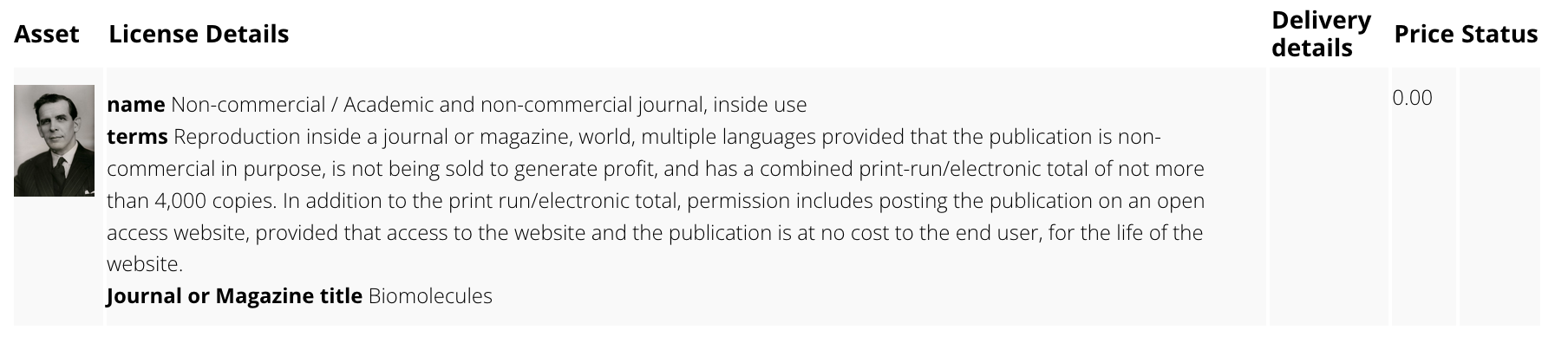
**

**1953:**

*X-ray chrystallography(sic) of DNA* 19 July 2015 Photo taken by Rosalind Franklin. Reproduced by Maria Evagorou, Sibel Erduran, Terhi Mäntylä This file is licensed under the [Creative Commons](https://en.wikipedia.org/wiki/en:Creative_Commons) [Attribution 4.0 International](https://creativecommons.org/licenses/by/4.0/deed.en) license.

https://commons.wikimedia.org/wiki/File:Fig-1-X-ray-chrystallography-of-DNA.gif

**1960**:

*Bundesarchiv Bild 183-1988-0830-010, Leipzig, Zoo, Elefantengehege*.jpg, image provided by German Federal Archive **Attribution:** Bundesarchiv, Bild 183-1988-0830-010 / Grubitzsch (geb. Raphael), Waltraud / CC-BY-SA 3.0, This file is licensed under the [Creative Commons](https://en.wikipedia.org/wiki/en:Creative_Commons) [Attribution-Share Alike 3.0 Germany](https://creativecommons.org/licenses/by-sa/3.0/de/deed.en) license. https://commons.wikimedia.org/wiki/File:Bundesarchiv_Bild_183-1988-0830-010,_Leipzig,_Zoo,_Elefantengehege.jpg

**1976:**

*St. Mary Magdalene Church*, *Taunton*, Date 4 April 2009, This file is licensed under the [Creative Commons](https://en.wikipedia.org/wiki/en:Creative_Commons) [Attribution-Share Alike 2.0 Generic](https://creativecommons.org/licenses/by-sa/2.0/deed.en) license.

Attribution: Ken Grainger

**1978:**

Author’s image of Gel electrophoresis with a ladder and RE digest: 1042 PCR.tif.

**1986:**

Cat *(Felis silvestris catus)* by David Orr

License Public Domain Mark 1.0

Uploaded 2022 Aug 13 by David Orr

Taxon *Felis silvestris catus* (Linnaeus 1758) Ragni & Randi 1986

**1990:**

**Figure 1[231] :** https://www.sciencedirect.com/science/article/pii/S1201971221002940?via%3Dihub

**Creative Commons Attribution-NonCommercial-No Derivatives License (CC BY NC ND)**

This article is published under the terms of the [Creative Commons Attribution-NonCommercial-No Derivatives License (CC BY NC ND)](http://creativecommons.org/licenses/by-nc-nd/4.0/).
For non-commercial purposes you may copy and distribute the article, use portions or extracts from the article in other works, and text or data mine the article, provided you do not alter or modify the article without permission from Elsevier. You may also create adaptations of the article for your own personal use only, but not distribute these to others. You must give appropriate credit to the original work, together with a link to the formal publication through the relevant DOI, and a link to the Creative Commons user license above. If changes are permitted, you must indicate if any changes are made but not in any way that suggests the licensor endorses you or your use of the work.

**License**


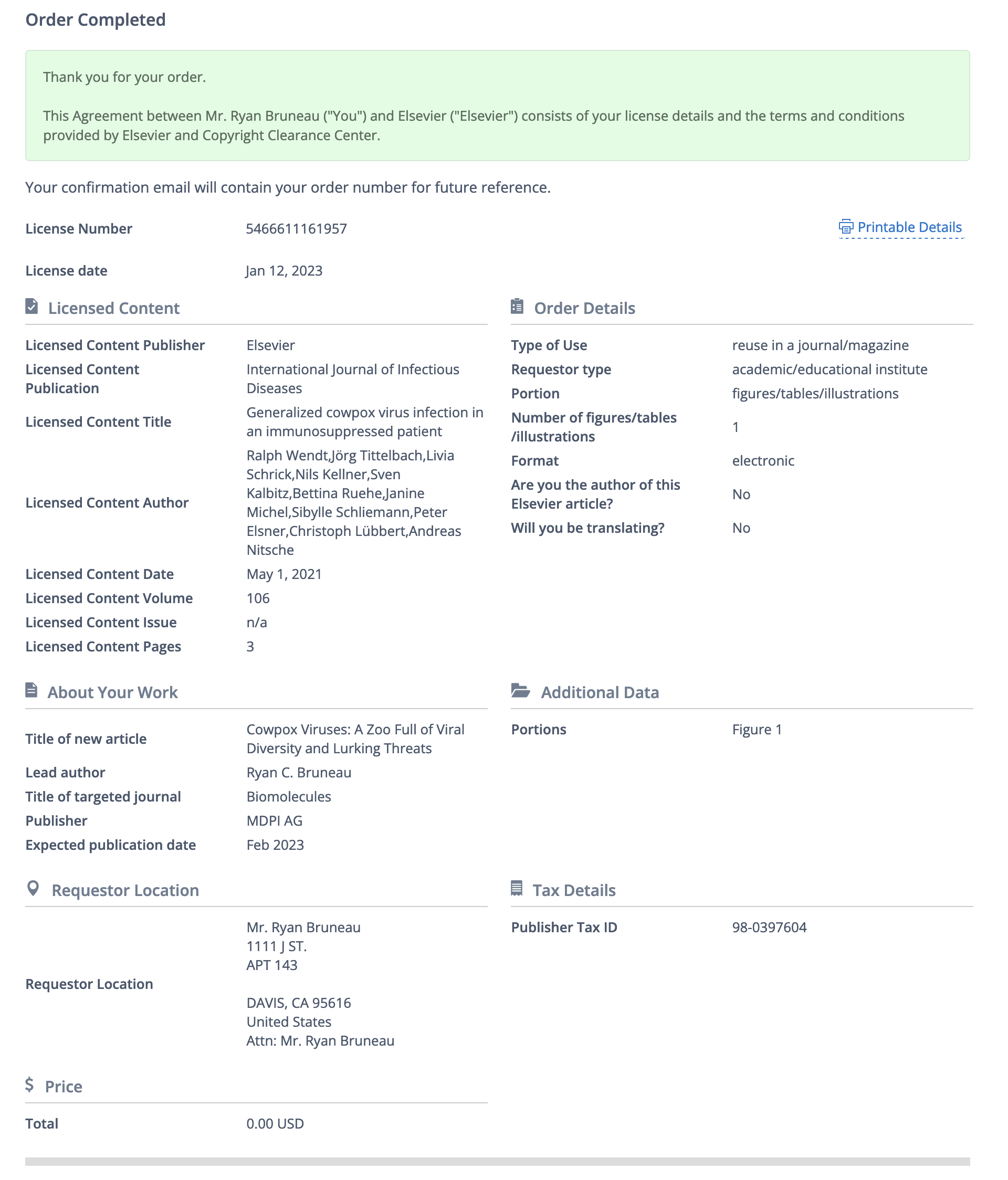


**2004:**

Reference[13] **Open Access content, rights:**

[**https://microbiologysociety.org/publication/past-issues/oceans/article/open-access-and-author-rights.html**](https://microbiologysociety.org/publication/past-issues/oceans/article/open-access-and-author-rights.html)

**2010s:**

Reference[156], “Seasonal recurrence of cowpox virus outbreaks in captive cheetahs (*Acinonyx jubatus*)”. adapted from figure 2. [Julia Stagegaard](https://pubmed.ncbi.nlm.nih.gov/?sort=pubdate&term=Stagegaard+J&cauthor_id=29121668)  [^1^](https://pubmed.ncbi.nlm.nih.gov/29121668/) , [Andreas Kurth](https://pubmed.ncbi.nlm.nih.gov/?sort=pubdate&term=Kurth+A&cauthor_id=29121668)  [^2^](https://pubmed.ncbi.nlm.nih.gov/29121668/)   [^3^](https://pubmed.ncbi.nlm.nih.gov/29121668/) , [Daniel Stern](https://pubmed.ncbi.nlm.nih.gov/?sort=pubdate&term=Stern+D&cauthor_id=29121668)  [^2^](https://pubmed.ncbi.nlm.nih.gov/29121668/) , [Piotr Wojciech Dabrowski](https://pubmed.ncbi.nlm.nih.gov/?sort=pubdate&term=Dabrowski+PW&cauthor_id=29121668)  [^4^](https://pubmed.ncbi.nlm.nih.gov/29121668/) , [Ann Pocknell](https://pubmed.ncbi.nlm.nih.gov/?sort=pubdate&term=Pocknell+A&cauthor_id=29121668)  [^5^](https://pubmed.ncbi.nlm.nih.gov/29121668/) , [Andreas Nitsche](https://pubmed.ncbi.nlm.nih.gov/?sort=pubdate&term=Nitsche+A&cauthor_id=29121668)  [^2^](https://pubmed.ncbi.nlm.nih.gov/29121668/)   [^3^](https://pubmed.ncbi.nlm.nih.gov/29121668/) , [Livia Schrick](https://pubmed.ncbi.nlm.nih.gov/?sort=pubdate&term=Schrick+L&cauthor_id=29121668)  [^2^](https://pubmed.ncbi.nlm.nih.gov/29121668/)   [^3^](https://pubmed.ncbi.nlm.nih.gov/29121668/)

**Copyright:** © 2017 Stagegaard *et al*. This is an open access article distributed under the terms of the [Creative Commons Attribution License](http://creativecommons.org/licenses/by/4.0/), which permits unrestricted use, distribution, and reproduction in any medium, provided the original author and source are credited.

**2011:**

Reference[129], **“**Chasing Jenner's Vaccine: Revisiting *Cowpox Virus”.* Carroll DS, Emerson GL, Li Y, Sammons S, Olson V, Frace M, *et al*. (2011) Chasing Jenner's Vaccine: Revisiting *Cowpox Virus* Classification. PLoS ONE 6(8): e23086. https://doi.org/10.1371/journal.pone.0023086 Classification This is an open-access article, free of all copyright, and may be freely reproduced, distributed, transmitted, modified, built upon, or otherwise used by anyone for any lawful purpose. The work is made available under the Creative Commons CC0 **public domain dedication**.

**2017:**

Reference^122^, “Cowpox virus: What's in a Name?”. [Matthew R Mauldin](https://pubmed.ncbi.nlm.nih.gov/?sort=pubdate&term=Mauldin+MR&cauthor_id=28486428)  [^1^](https://pubmed.ncbi.nlm.nih.gov/28486428/)   [^2^](https://pubmed.ncbi.nlm.nih.gov/28486428/) , [Markus Antwerpen](https://pubmed.ncbi.nlm.nih.gov/?sort=pubdate&term=Antwerpen+M&cauthor_id=28486428)  [^3^](https://pubmed.ncbi.nlm.nih.gov/28486428/) , [Ginny L Emerson](https://pubmed.ncbi.nlm.nih.gov/?sort=pubdate&term=Emerson+GL&cauthor_id=28486428)  [^4^](https://pubmed.ncbi.nlm.nih.gov/28486428/) , [Yu Li](https://pubmed.ncbi.nlm.nih.gov/?sort=pubdate&term=Li+Y&cauthor_id=28486428)  [^5^](https://pubmed.ncbi.nlm.nih.gov/28486428/) , [Gudrun Zoeller](https://pubmed.ncbi.nlm.nih.gov/?sort=pubdate&term=Zoeller+G&cauthor_id=28486428)  [^6^](https://pubmed.ncbi.nlm.nih.gov/28486428/) , [Darin S Carroll](https://pubmed.ncbi.nlm.nih.gov/?sort=pubdate&term=Carroll+DS&cauthor_id=28486428)  [^7^](https://pubmed.ncbi.nlm.nih.gov/28486428/) , [Hermann Meyer](https://pubmed.ncbi.nlm.nih.gov/?sort=pubdate&term=Meyer+H&cauthor_id=28486428)  [^8^](https://pubmed.ncbi.nlm.nih.gov/28486428/)

No special permission is required to reuse all or part of article published by MDPI, including figures and tables. For articles published under an open access Creative Common CC BY license, any part of the article may be reused without permission provided that the original article is clearly cited. Reuse of an article does not imply endorsement by the authors or MDPI.

**2022:**

File:*Cowpox virus*.jpg Description

English: Electron micrograph of three cowpox virus particles

Date 7 May 2017, 07:03:56 Author [Dr Graham Beards](https://commons.wikimedia.org/wiki/User:Graham_Beards), This file is licensed under the [Creative Commons](https://en.wikipedia.org/wiki/en:Creative_Commons) [Attribution-Share Alike 4.0 International](https://creativecommons.org/licenses/by-sa/4.0/deed.en) license.

**Figure 3 Hosts of fully sequenced CPXV isolates**

Cat (*Felis silvestris catus)* by David Orr

License Public Domain Mark 1.0

Uploaded 2022 Aug 13 by David Orr

Taxon *Felis silvestris catus* (Linnaeus 1758) Ragni & Randi 1986

Human (*Homo sapiens sapiens*) by NASA

License Public Domain Mark 1.0

Uploaded 2022 Aug 13 by Yan Wong

Taxon *Homo sapiens sapiens* Linnaeus 1758

Image modified for use by authors.

Rat *(Rattus norvegicus)* by Ryan Cupo

License CC0 1.0 Universal Public Domain Dedication

Uploaded 2022 Aug 13 by Ryan Cupo

Taxon *Rattus norvegicus* (Berkenhout 1769)

Bank vole (*Myodes glareolus*)

License CC0 1.0 Universal Public Domain Dedication

Uploaded 2022 Aug 13 by Callum Le Lay

Taxon *Myodes glareolus*

Common Marmoset (*Callithrix jacchus*) by Yan Wong from drawing by T. F. Zimmermann

License CC0 1.0 Universal Public Domain Dedication

Uploaded 2022 Aug 13 by Yan Wong

Taxon *Callithrix jacchus* (Linnaeus 1758)

Banded Mongoose *(Mungos mungo)* by Birgit Lang

License CC0 1.0 Universal Public Domain Dedication

Uploaded 2022 Aug 13 by Birgit Lang

Taxon *Mungos mungo* (Gmelin 1788)

Raccoon *(Procyon lotor)* by Steven Traver

License CC0 1.0 Universal Public Domain Dedication

Uploaded 2022 Aug 13 by Steven Traver

Taxon *Procyon lotor* (Linnaeus 1758)

Beaver *(Castor canadensis)*

License Public Domain Mark 1.0

Uploaded 2022 Aug 13 by xgirouxb

Taxon *Castor canadensis* Kuhl 1820

Cheetah *(Acinonyx jubatus)* by Gabriela Palomo-Munoz

License Attribution-NonCommercial 3.0 Unported

Uploaded 2022 Aug 13 by Gabriela Palomo-Munoz

Taxon *Acinonyx jubatus* (Schreber 1775)

Boa (*Boa constrictor*) by Becky Barnes

License CC0 1.0 Universal Public Domain Dedication

Uploaded 2022 Aug 13 by Becky Barnes

Taxon *Boa constrictor* LINNAEUS 1758: 215

All above photos for Figure 3 sourced from <https://beta.phylopic.org/>

Common Vole (*Microtus arvalis*) Picture

(c) Aleksandar, some rights reserved (CC BY-SA), uploaded by Aleksandar

<https://www.inaturalist.org/guide_taxa/889037>

<https://creativecommons.org/licenses/by-sa/2.0/>

Image modified for use by authors.

Alpaca (*Lama pacos*) Public Domain

<https://publicdomainvectors.org/en/free-clipart/Alpaca/62010.html>

Image modified for use by authors.

Jaguarundi (*Herpailurus yagouaroundi*) Public Domain

Description Français: Jaguarundi (*Puma yagouaroundi*) au zoo de Pont-Scorff.

Date 17 August 2015

Source Own work

Author Vassil

<https://commons.wikimedia.org/wiki/File:Puma_yagouaroundi.jpg>

Image modified for use by authors.

Prairie Dog (genus: *Cynomys*) Public Domain

<https://www.publicdomainpictures.net/en/view-image.php?image=215624&picture=prairie-dog>

Image modified for use by authors.

Mara (genus: *Dolichotis)*

Free to use: https://www.pexels.com/license/

<https://www.pexels.com/photo/photo-of-a-patagonian-mara-on-brown-soil-5840623/>

Image modified for use by authors.

Cotton Tailed Tamarin (*Saguinus oedipus*)

CC0 Public Domain Free for personal and commercial use. No attribution required. <https://pxhere.com/en/photo/817787>

Image modified for use by authors.

Horse (*Equus ferus caballus*)

<https://www.flaticon.com/free-icon/jumping-horse-silhouette-facing-left-side-view_33348>

###### <a href="https://www.flaticon.com/free-icons/horse" title="horse icons">Horse icons created by Freepik - Flaticon</a> Flaticon license

Free for personal and commercial use with attribution

Elephant (Genus: *Elephas*)

License: Public domain

https://publicdomainvectors.org/en/free-clipart/Black-elephant-vector-image/73498.html
