# Supplementary figures and images for: Cowpox Viruses: A Zoo Full of Viral Diversity and Lurking Threats
Source: Biomolecules. 2023 Feb 8;13(2):325. doi: 10.3390/biom13020325 (PMC9953750; doi:10.3390/biom13020325)

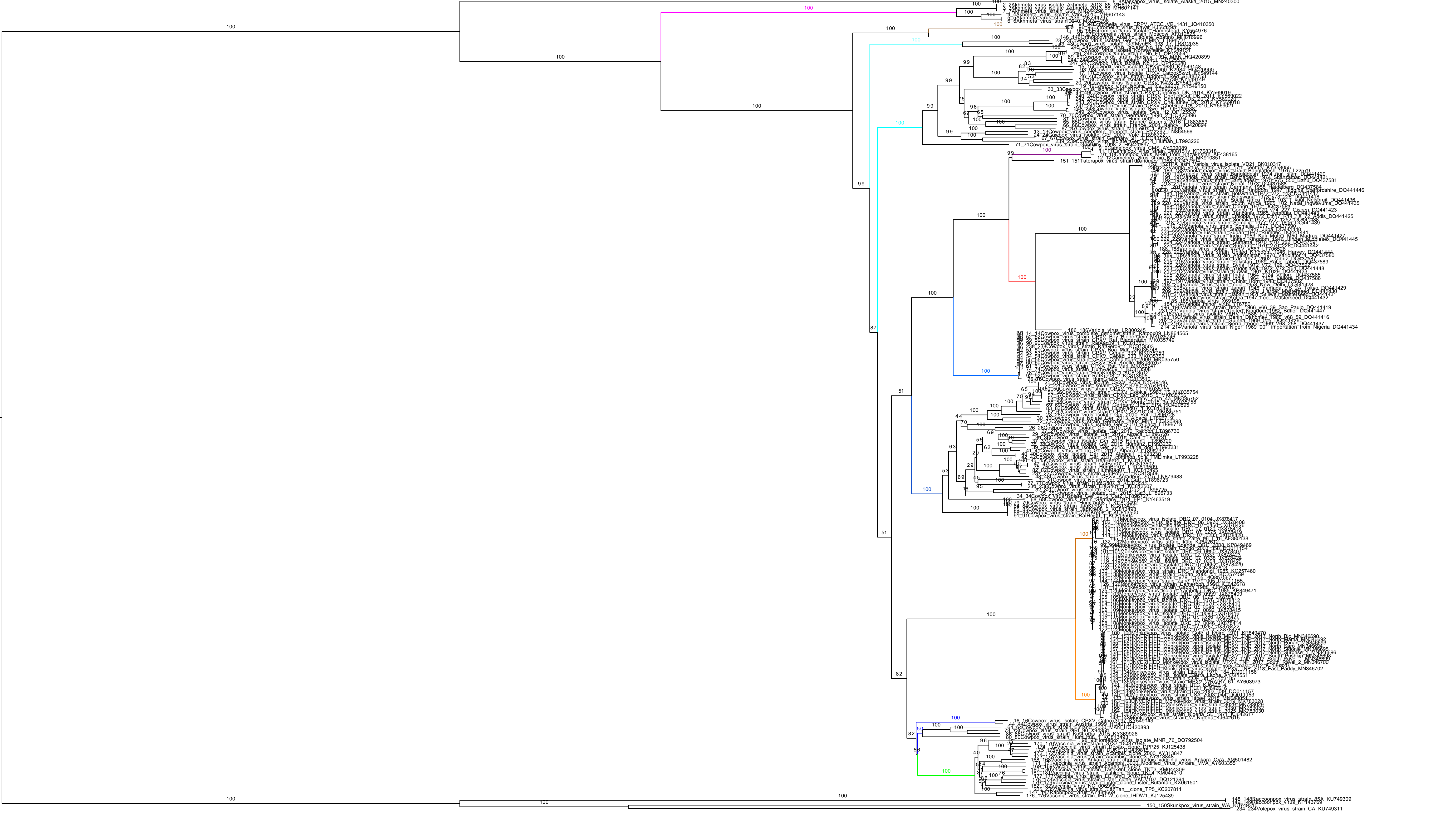

Supplement: Supplementary file 1 [file biomolecules-13-00325-s001.zip › Supplementary file S1 full_OPV_phylogeny.pdf]
